# Plasma exosomes impair microglial degradation of α‐synuclein through V‐ATPase subunit V1G1

**DOI:** 10.1111/cns.14738

**Published:** 2024-05-03

**Authors:** Yunna Li, Yiming Wang, Liang Kou, Sijia Yin, Xiaosa Chi, Yadi Sun, Jiawei Wu, Zongjie Jin, Qiulu Zhou, Wenkai Zou, Tao Wang, Yun Xia

**Affiliations:** ^1^ Department of Neurology, Union Hospital, Tongji Medical College Huazhong University of Science and Technology Wuhan China; ^2^ Department of Neurology, The Central Hospital of Wuhan, Tongji Medical College Huazhong University of Science and Technology Wuhan China

**Keywords:** exosome, lysosome, microglia, V‐ATPase, α‐synuclein

## Abstract

**Introduction:**

Microglia are the main phagocytes in the brain and can induce neuroinflammation. Moreover, they are critical to alpha‐synuclein (α‐syn) aggregation and propagation. Plasma exosomes derived from patients diagnosed with Parkinson's disease (PD‐exo) reportedly evoked α‐syn aggregation and inflammation in microglia. In turn, microglia internalized and released exosomal α‐syn, enhancing α‐syn propagation. However, the specific mechanism through which PD‐exo influences α‐syn degradation remains unknown.

**Methods:**

Exosomes were extracted from the plasma of patients with PD by differential ultracentrifugation, analyzed using electron microscopy (EM) and nanoparticle flow cytometry, and stereotaxically injected into the unilateral striatum of the mice. Transmission EM was employed to visualize lysosomes and autophagosomes in BV2 cells, and lysosome pH was measured with LysoSensor Yellow/Blue DND‐160. Cathepsin B and D, lysosomal‐associated membrane protein 1 (LAMP1), ATP6V1G1, tumor susceptibility gene 101 protein, calnexin, α‐syn, ionized calcium binding adaptor molecule 1, and NLR family pyrin domain containing 3 were evaluated using quantitative polymerase chain reaction or western blotting, and α‐syn, LAMP1, and ATP6V1G1 were also observed by immunofluorescence. Small interfering ribonucleic acid against V1G1 was transfected into BV2 cells and primary microglia using Lipofectamine® 3000. A PD mouse model was established via injection with 1‐methyl‐4‐phenyl‐1,2,3,6‐tetrahydropyridine (MPTP) into mice. A lentiviral‐mediated strategy to overexpress ATP6V1G1 in the brain of MPTP‐treated mice was employed. Motor coordination was assessed using rotarod and pole tests, and neurodegeneration in the mouse substantia nigra and striatum tissues was determined using immunofluorescence histochemical and western blotting of tyrosine hydroxylase.

**Results:**

PD‐exo decreased the expression of V1G1, responsible for the acidification of intra‐ and extracellular milieu. This impairment of lysosomal acidification resulted in the accumulation of abnormally swollen lysosomes and decreased lysosomal enzyme activities, impairing lysosomal protein degradation and causing α‐syn accumulation. Additionally, V1G1 overexpression conferred the mice neuroprotection during MPTP exposure.

**Conclusion:**

Pathogenic protein accumulation is a key feature of PD, and compromised V‐type ATPase dysfunction might participate in PD pathogenesis. Moreover, V1G1 overexpression protects against neuronal toxicity in an MPTP‐based PD mouse model, which may provide opportunities to develop novel therapeutic interventions for PD treatment.

## Graphical abstract

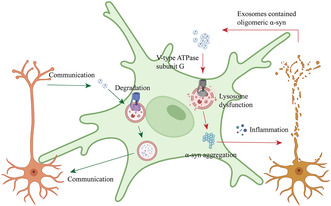

## 1 INTRODUCTION

Parkinson's disease (PD) is a common disabling progressive neurodegenerative disease characterized by the loss of dopaminergic neurons in the pars compacta of the substantia nigra (SNPC) as well as the extensive aggregation of α‐synuclein (α‐syn) in the injured neurons and the formation of Lewy bodies.^1^ The relationship between α‐syn and microglia, the central nervous system (CNS) resident immune cells, was in the spotlight of PD research. Microglia engulf neuron‐released α‐syn into autophagosomes for degradation via Toll‐like receptor (TLR) 4‐nuclear factor κB‐p62‐mediated selective autophagy, contributing to clearance of pathologic α‐syn and alleviating neurodegeneration.^2^ Interestingly, the fibrillar α‐syn burden within microglia can be jointly ‘shared’ by distribution through tunneling nanotubes, which transfer a‐syn from overloaded microglia to neighboring naive microglia where the a‐syn cargo got degraded rapidly and effectively.^3^ Intercellular transfer of fibrillar α‐syn attenuates the inflammatory profile of microglia and protects them from cytotoxicity and cell death. However, microglia reportedly play a pivotal role in α‐syn transmission in PD.^4^, ^5^ Microglia internalize the exosomal α‐syn through TLR 2 located in the membrane, enhancing the propagation of α‐syn from microglia to the neuron.^4^ Recent studies have shown that exosomes act as ‘vehicle vesicles’ to traffic pathological α‐syn, and α‐syn found in exosomes in the cerebrospinal fluid (CSF) of patients with PD and dementia with Lewy bodies induces α‐syn aggregation.^6^, ^7^ Additionally, when exposed to human α‐syn performed fibrils, microglia release exosomes containing α‐syn, which are fully capable of inducing protein aggregation in the recipient neurons. The aforementioned study reported that microglia act as a double‐edged sword in the progression of PD. Although the mechanism by which microglia degrade and propagate α‐syn has been described, the role of lysosomes in microglia remains unknown.

V‐ATPases are multi‐subunit complexes located on the lysosomal membrane, composed of the cytosolic catalytic V1 part and the membrane‐bound V0 part. V‐ATPases act as proton pumps to pump protons into the lysosomal lumen.^6^ The establishment and maintenance of an optimal pH in the lysosomal lumen are highly dependent on the V‐ATPases. The ‘steady‐state’ acidic pH is required for the optimal activities of most hydrolytic enzymes and the function of degradation, nutrient sensing, and cargo loading in the lysosome. Studies have shown that the V‐ATPases‐dependent lysosomal pH and maintenance of lysosomal physiological functions are tightly associated with neurodegeneration diseases,^7^ such as Alzheimer's disease (AD), PD, and lysosomal storage diseases,^8^, ^9^ where lysosomes can degrade and clear pathological protein aggregates.^10^ Although V‐type ATPase subunit G (V1G1), one of the subunits of V‐ATPases, can regulate the activity of V‐ATPases and the biological generation of lysosomes,^11^, ^12^ the specific role of V1G1 in the degradation of exosomal α‐syn by microglia remains unknown.

In this work, we treated microglia with plasma‐derived exosomes from patients with PD (PD‐exo) to explore their effect on microglial lysosomes and the role of lysosomal V1G1 in the degradation of exosomal α‐syn by microglia.

## 2 MATERIALS AND METHODS

### 2.1 Samples, isolation, and characterization of exosomes

Human plasma samples were collected from Wuhan Union Hospital after obtaining informed consent from patients and approval from the ethics committee. PD patients with disease duration over 5 years and age over 60 years and age‐matched counterparts were included in this study according to the 2015 Movement Disorder Society Clinical Diagnostic Criteria for Parkinson's disease. Exosomes were extracted from plasma by differential ultracentrifugation as described previously with slight modifications.^13^ Briefly, plasma was diluted with an equal volume of phosphate‐buffered saline (PBS) and then centrifuged for 45 min at 12,000 *g*. The supernatant was centrifuged for 2 h at 110,000 *g* in Beckman ultracentrifuge tubes. The resuspended pellets in a large volume PBS were centrifuged again for 70 min at 110,000*g* following filtered through a 0.22 m filter. The exosome pellet was resuspended in 50 to 100 μL PBS or RIPA lysis buffer stored at −80°C for further experiments. All centrifugations were performed at 4°C.

The morphology, particle size, and concentration characteristic of the plasma‐derived exosome were analyzed using electron microscopy (EM) and NanoFCM. Ten microliters of exosome sample was added dropwise to the copper grid for 1 min. After the floating liquid was removed by filter paper, add dropwise 10 μL of uranium dioxide acetate to the copper grid for 1 min. Dried at room temperature for several minutes and the exosomes were observed subsequently at 100 kV under electron microscopy. Dilute the exosome preparation to a suitable concentration. The isolated exosomes were detected with the NanoFCM following the instrument calibrated with the standard sample.

### 2.2 Mice

Male C57BL/6 mice, 8 weeks old, were randomly divided into four groups: the mice in the Con‐exo group were stereotaxically injected with the healthy control plasma‐derived Con‐exo in the unilateral striatum (+ 0.3 anterior–posterior, +2.0 medial–lateral, −2.8 dorsal–ventral), while the mice in the PD‐exo group were injected with the PD patients' plasma‐derived PD‐exo as described previously. The mice in the MPTP group were continuously intraperitoneally administered with MPTP solution (30 mg/kg/day) for 7 days, while the mice in the control group were administered with an equal volume of saline. After 1 to 2 weeks of modeling, the motor function of these mice was tested, and then, the mice were sacrificed for brain serial sectioning for further experiments.

### 2.3 Intracerebral injection of lentiviral particles

Male C57BL/6 mice, 8 weeks old, were randomly divided into three groups: LV‐con + WT group, LV‐con + MPTP group, and LV‐V1G1 + MPTP group. Lentiviral particles were injected into the unilateral striatum (+0.3 anterior–posterior, +2.0 medial–lateral, −2.8 dorsal–ventral). After lentiviral vector‐mediated V1G1 gene overexpression at 1 week, obvious expression of V1G1 was observed. The mice in the MPTP group were continuously intraperitoneally administered with MPTP solution (30 mg/kg/day) for 7 days, while the mice in the control group were administered with an equal volume of saline. After 1 to 2 weeks of modeling, the motor function of these mice was tested, and then, the mice were sacrificed for brain serial sectioning for further experiments.

The motor function of the mice was tested 6–7 days after the last MPTP injection. In the rotarod test, the mice were trained to stay on the rotarod for 3 consecutive days before the test. When officially tested, the latency to fall in the rotarod test was measured at a speed accelerating from 4 to 40 rpm in 2 min. The mice were tested 3 times with a rest of 5 min between each trial. In the pole test, the mice were placed face‐up near the top of a rough wooden pole (15 mm in diameter and 40 cm in height), and the time it took to reach the floor was recorded. After 3 days of learning adaptation, each mouse was finally administered three formal tests. The recorded values in all tests were averaged for each mouse and then used for statistical analysis.

### 2.4 Immunofluorescence

BV2 cells cultured and mounted onto coverslips in 24‐well plates were fixed with 4% paraformaldehyde (PFA) in PBS for 15 min at room temperature (RT). Cells were washed 3 times with PBS and were then permeabilized by 0.5% Triton X‐100 for 5 min at RT. Cells were washed 3 times with PBS, and 1% bovine serum albumin (BSA) in PBS was then used to block the nonspecific antibody binding sites for 30 min. Subsequently, cells were incubated with primary antibodies overnight at 4°C. Cells were washed 3 times with PBS on the following day and then incubated for anti‐mouse or anti‐rabbit secondary antibodies conjugated to Alexa Fluor® 488 or 647 (AntGene, Code No. ANT044, ANT032S) for 1 h at RT, followed by three short wash steps. Cells were stained with cellular nuclei dye DAPI for 3 min, then followed by 3 times short wash. After cells were immersed with ProLong™ Diamond Antifade Mountant solution (Invitrogen™, Catalog no: P36970) to avoid fluorescence quenching, image acquisitions were then performed by a Nikon A1‐Si confocal microscope with identical settings.

Mice were sacrificed for serial frozen sections of brain tissue. The frozen slides then undergo a series of fixation, permeabilization, blocking, and staining steps as described previously with slight modifications. Similarly, image acquisitions of brain tissue slides were performed by a Nikon A1‐Si confocal microscope with identical settings. The primary antibodies for immunofluorescence are listed as follows: rabbit antibody to ATP6V1G1 (1;100, 16143‐1‐AP, Proteintech), rat antibody to LAMP1 (1:100,1D4B, DSHB), and rabbit antibody to LAMP1 (1:100, ab208943, Abcam); mouse antibody α‐syn (1:300, MA5‐12272, Thermo Fisher; 1:100, sc12767, Santa Cruz Biotechnology), rabbit antibody to TH (1:200, ab112, Abcam), and rabbit antibody to IBA1 (1:500, 019‐19741, Wako).

### 2.5 Transmission electron microscopy

Transmission electron microscopy (TEM) was employed to visualize lysosomes and autophagosomes in BV2 cells treated with PBS, Con‐exo, and PD‐exo. Samples were processed by Wuhan Pinofei Biotechnology Co. In brief, BV2 cells were blown down and transferred into 10‐mL sterile tubes, then centrifuged at 800 rpm for 5 min. The most volume of the supernatant was discarded, while 1 mL volume of supernatant was retained. Cells then were gently blown apart and transferred into 1.5‐mL EP tubes, and the supernatant was aspirated after settling vertically for 1 h. 2.5% glutaraldehyde in cacodylate buffer was used to fix the cell precipitation. The preparation sample was mounted on a 150‐mesh hexagonal copper grid and visualized by transmission electron microscope. The morphology characteristic of lysosomes was analyzed by FIJI software.

### 2.6 Lyso‐Tracker Red staining and live cell imaging

BV2 cells were planted on 15‐mm confocal dishes and were treated with PD‐exo and Con‐exo for 24 h after adhering to the wall. Lyso‐Tracker Red (Beyotime) was introduced to the cell culture medium at 1:20,000 dilution to make a 50 nM Lyso‐Tracker Red staining working solution. Following the staining working solution pre‐incubated at 37°C, BV2 cells treated with exosomes were incubated for 30 min in the working solution at 37°C. For this experiment, the Hoechst 33342 was used to stain the nucleus under live cell conditions. BV2 cells were then immediately imaged by a confocal microscope in living conditions. All images were acquired under uniform microscope settings. The integrated fluorescence intensity was analyzed and processed by ImageJ software.

### 2.7 Lysosomal pH measurement

The lysosomal pH was measured as described previously^14^, ^15^ with slight modification with LysoSensor Yellow/Blue DND‐160 (L7545, Invitrogen). BV2 cells were treated with PD‐exo or Con‐exo for 24 h. 2 μM LysoSensor Yellow/Blue DND‐160 was introduced to the treated BV2 cells for 10 min at RT. Cells were then transferred to black 96‐well plates after three rinses with PBS and resuspension. The lysosensor fluorescence intensity of BV2 cells in black 96‐well plates was recorded with a plate reader (BioTek) at 340 and 380 nm upon excitation with 527 nm upon emission. To obtain the lysosomal pH value, the 340 nm/380 nm ratio was interpolated to a calibration curve. The calibration curve was obtained through a series of pH calibration buffers (range from 3.5 to 7.0) containing BV2 cells, which is stained with the DND‐160. The 340/380 value was measured for these pH calibration buffers. The lysosomal pH calibration curve can be established according to the 340/380 value and corresponding pH value.

### 2.8 Culture, siRNA transfection of cells

Primary rat cortical microglia were obtained from 1‐day‐old Sprague–Dawley rats. Briefly, dissected cortices without meninges were separated into smaller pieces, and trypsin was added. After digestion, single‐cell suspensions were generated, and the mixtures were centrifuged at 300 *g* for 10 min. The cell pellet was resuspended with fresh medium and plated at 100,000 cells/well on poly‐D‐lysine‐coated 24‐well plates (Solarbio, P8140). The cells are cultured for about 14 to 16 days and digested with 2 to 3 mL of 0.05% trypsin, while observing, gently shake the culture bottle until the microglia attached to the astrocytes detach, transfer the digestion solution containing floating microglia into a 10‐mL centrifuge tube, and immediately terminate the digestion with a complete culture medium. Centrifuge tube trim, 1000 r/min, centrifuge for 5 min, and discard supernatant. The cell suspension was then blown into a quantitative complete culture and inoculated into a pre‐coated 24‐well culture plate with a covered glass slide and cultured in a CO2 constant temperature cell incubator (37 degrees). After 24‐h growth, the culture medium was sucked to remove the unadherent oligodendrocytes, and the culture was continued with a complete culture medium.

BV2 cell line, a mouse‐derived immortalized microglial cell line, was purchased from the BMCR national biomedical cell line resource center. Cells were cultured in DMEM including 4.5 g/L glucose (Gibco) supplemented with 10% of fetal bovine serum (FBS) at 37°C, gassed with 5% CO2. To downregulated ATP6V1G1(V1G1) gene, three pairs of siRNA duplex against mouse V1G1 were designed and synthesized (Tsingke Biotechnology). siRNA against V1G1 was transfected into BV2 cells in parallel to siRNA scramble (control siRNA) by using Lipofectamine® 3000 (Invitrogen) according to the manufacturer's instructions. After incubated for 48 h with siRNA, BV2 cells were treated with PD‐exo for 24 h for western blot analysis.

### 2.9 Real‐time RT‐PCR

BV2 cells were lysated by TRIzon reagent (Cwbio, Code No. CW0580s) for total RNA isolation. Then, lysates above were used to isolate and purify with the Ultrapure RNA Kit (Cwbio, CW0581M) according to the manufacturer's protocol for total RNA. The obtained total RNA was reversed with HiScript III 1st Strand cDNA Synthesis Kit (Vazyme, Code No. R312‐01) for cDNA. The target genes' mRNA levels were analyzed by real‐time RT‐PCR using a StepOnePlusTM system according to the cDNA. The sequences of the specific primers for target genes are listed below:

Cathepsin B: forward (5′‐TCCTTGATCCTTCTTTCTTGCC‐3′) and reverse (5′‐ACAGTGCCACACAGCTTCTTC‐3′);

Cathepsin D: forward (5′‐GCTTCCGGTCTTTGACAACCT‐3′) and reverse (5′‐CACCAAGCATTAGTTCTCCTCC‐3′);

LAMP1: forward (5′‐CAGCACTCTTTGAGGTGAAAAAC‐3′) and reverse (5′‐ACGATCTGAGAACCATTCGCA‐3′);

ATP6V1G1: forward (5′‐CCCAGGCTGAAATTGAACAGT‐3′) and reverse (5′‐TTCTGGAGGACGGTCATCTTC‐3′). The data of real‐time PCR were analyzed using the value 2−△△Ct. Mouse β‐actin was used as the reference genes.

### 2.10 Western blotting

Cells were rinsed with PBS for two washes and then lysed in RIPA buffer (Beyotime, P0013C) supplemented with a protease and phosphatase inhibitor cocktail (Servicebio, G2006, G2007) on ice for 20 min. The lysates were centrifuged at 12,000 g for 15 min at 4°C. The supernatant, which is the whole cell lysate, was supplemented with 5× sodium dodecyl sulfate (SDS) loading buffer (1:5 dilution, Servicebio, G2013‐1ML) and then boiled at 95°C for 10 min. After separated on SDS‐PAGE gels, the protein samples were transferred to PVDF membrane. Membranes were then blocked by 5% milk for 1 h and washed for three times. Subsequently, the protein‐loaded PVDF membrane was incubated by primary antibody overnight and corresponding horseradish peroxidase (HRP)‐conjugated secondary antibody for 1 h (1:10,000 dilution, ANT019, ANT020, Antgene), respectively. The protein bands were lastly visualized using the ECL immunoblot chemiluminescence system and analyzed by ImageJ software.

For the western blot experiment, the following primary antibodies were used: rabbit antibody to TSG101 (1:1000, 28283‐1‐AP, Proteintech); rabbit antibody to calnexin (1:1000, 10427‐2‐AP, Proteintech); rabbit antibody to ATP6V1G1 (1;100, 16143‐1‐AP, Proteintech), rat antibody to LAMP1 (1:100, 1D4B, DSHB), rabbit antibody to LAMP1 (1:100, ab208943, Abcam); rabbit antibody to cathepsin D (1:2000, ab75852, Abcam); mouse antibody α‐syn (1:300, MA5‐12272, Thermo Fisher); rabbit antibody α‐syn (1:100, ab138501, Abcam), rabbit antibody to IBA1 (1:1000, ab178846, Abcam); rabbit antibody NLRP3 (1:1000, ab263899, Abcam); mouse antibody to beta‐actin (1:1000, 66009‐1‐Ig, Proteintech); mouse antibody to GAPDH (1:1000, 60004‐1‐Ig, Proteintech); rabbit antibody to TH (1:200, ab112, Abcam). Original uncropped and unadjusted immunoblots, including molecular size markers, were provided (Figures S1–S8).

### 2.11 Statistical analysis

All values were expressed as mean ± SEM of at least three independent differentiations. Statistical comparisons between the two groups were performed with Student's *t*‐tests. For the analysis of more than two groups, a one‐way ANOVA test was performed. All statistics were calculated using GraphPad Prism software (San Diego, CA, USA), and a value of *p* < 0.05 was considered statistically significant.

## 3 RESULTS

### 3.1 Plasma exosomes derived from patients with PD carry more pathogenic α‐syn

**FIGURE 1:**
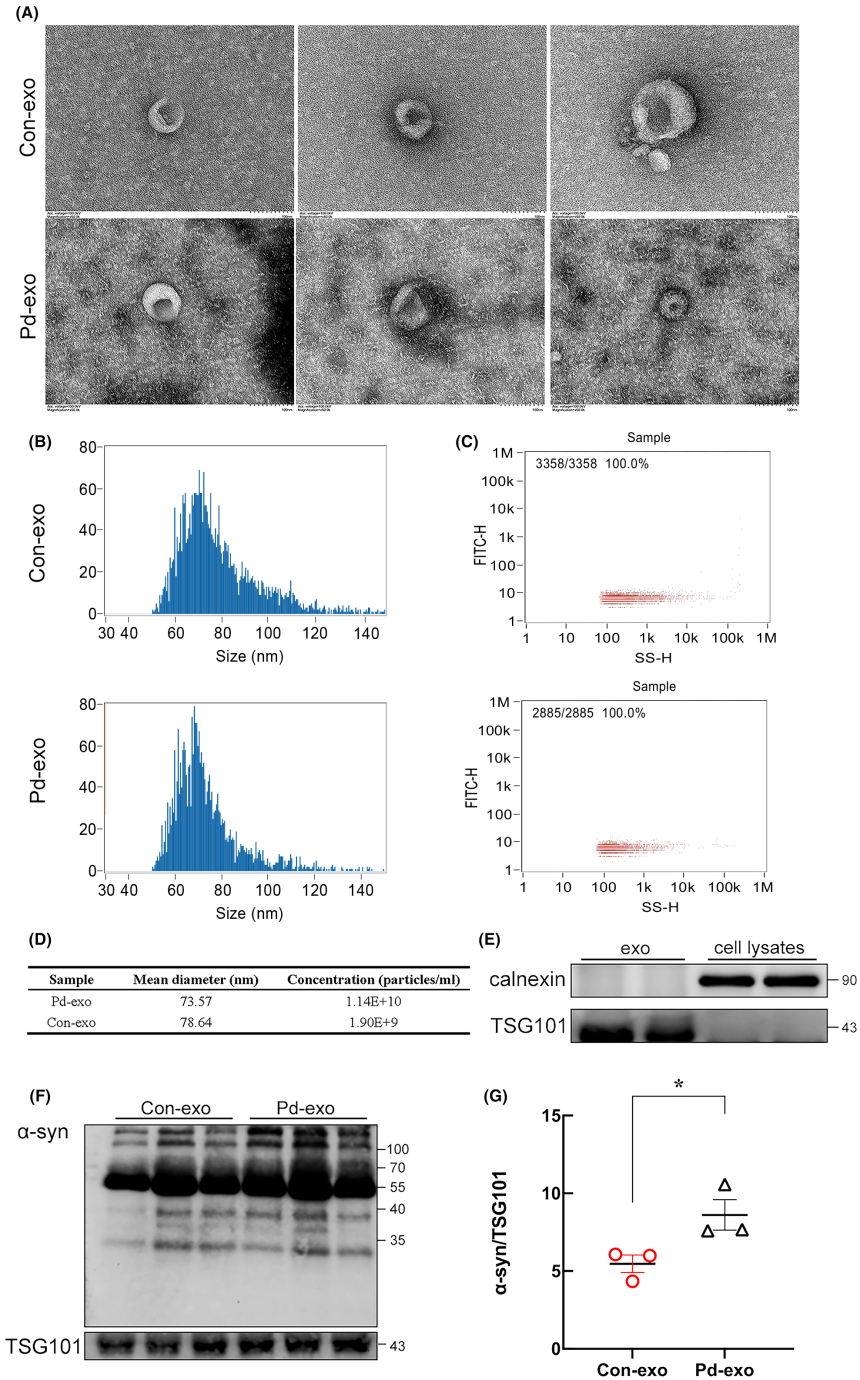
Increased α‐synuclein egress in plasma exosomes derived from patients with PD. (A) Representative transmission electron microscopy observation of exosomes isolated from human plasma, Scale bar = 100 nm. (B) Nanotracker particle analysis determined the size of exosomes isolated from human plasma. (C) Nanotracker particle analysis determined the concentration of exosomes isolated from human plasma. (D) Representative data on particle size and concentration of exosomes. (E) Western blot shows the presence of exosomal marker TSG101 and the absence of negative marker calnexin in plasma‐derived exosomes. (F) Immunoblots show the levels of oligomeric and monomeric exosomal α‐syn expression. (G) The quantitative data of oligomeric exosomal α‐syn. Bolts were probed for TSG101 as a loading control. *p < 0.05.

The exosome‐rich fraction was purified from the plasma of patients with PD and healthy controls using differential ultracentrifugation (Table 1). PD‐exo and exosomes obtained from the plasma of healthy controls (Con‐exo) were confirmed by transmission electron microscope (TEM) study, nanoparticle flow cytometry (NanoFCM), and western blotting. Representative TEM images of the exosomes of patients depict typical membrane ‘cup and plate’ structures (Figure 1A). The NanoFCM results showed that the diameters of the two groups of plasma exosomes concentrated at approximately 75 nm (Figure 1B). Interestingly, the exosome concentration of PD‐exo was higher than that of Con‐exo (Figure 1C,D). Western blotting confirmed that TSG101 was expressed in the exosomal samples but not in the supernatants of cell lysates (Figure 1E). Moreover, calnexin, an endoplasmic reticulum protein, was only detectable in cell lysates (Figure 1E).

**TABLE 1:** Clinical characteristics of patients with Parkinson's disease included in the study.

Subject No.	Age (year)	Gender (M/F)	Age at onset	UPDRS III	H&Y
1	78	M	71	41	3.0
2	55	M	53	28	1.5
3	59	F	49	28	2.5
4	68	F	38	58	4.0
5	69	F	67	18	2.0
6	73	M	52	32	3.0
7	53	M	48	28	1.5
8	48	M	41	26	2.0
9	68	F	54	30	2.5
10	77	M	57	54	4.0
11	65	F	60	16	1.5
12	36	F	25	49	3.0

As α‐syn may be central to PD pathophysiology, the concentrations and forms of total α‐syn in plasma‐derived exosomes were determined by immunoblotting. The results showed that PD‐exo contains higher concentrations of α‐syn oligomer when compared to similar preparations of Con‐exo (Figure 1F,G). However, the underlying mechanisms for the effect of exosomes containing toxic α‐syn oligomers remain unknown.

### 3.2 Tandem mass tag (TMT)‐based quantitative proteomic analysis reveals the regulation of plasma exosomes on the protein degradation of microglia

**FIGURE 2:**
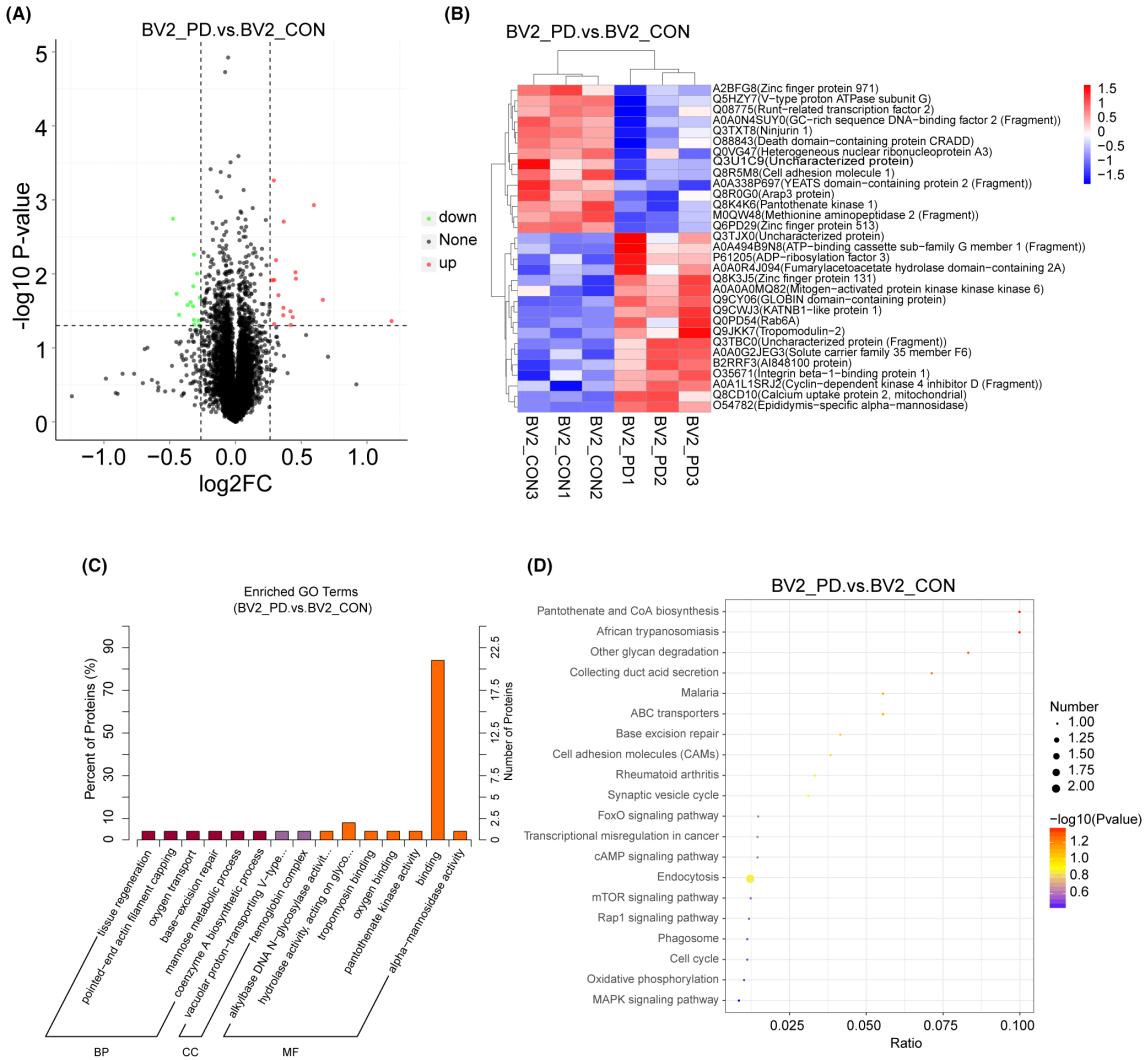
Quantitative proteome analysis of plasma exosome‐treated microglia. (A) Differential protein volcano map analysis of PD‐exo‐treated microglia versus Con‐exo‐treated microglia. (B) The hierarchical clustering heat map indicates the candidates for differentially expressed proteins in exosome‐treated microglia. (C) GO analysis of cellular components of all identified proteins in exosomes‐treated microglia. (D) KEGG analysis of cellular components of all identified proteins in exosomes‐treated microglia.

To assess the molecular mechanism of exosomes containing toxic α‐syn oligomers on the microglia, TMT‐based quantitative proteomic analysis was used. Volcano plot, hierarchical cluster, gene ontology (GO), and Kyoto Encyclopedia of Genes and Genomes (KEGG) pathway analysis were conducted. TMT‐based quantitative proteomics analysis found that 6700 quantifiable proteins were identified, and 31 (17 upregulated and 14 downregulated) differentially expressed proteins were identified in the PD‐exo‐treated and Con‐exo‐treated microglia groups (Figure 2A,B).

GO analysis of cellular components indicated that most proteins were annotated as vacuolar proton‐transporting V‐type ATPase complexes (Figure 2C). Regarding biological progress, the proteins mediated ‘tissue regeneration’ and ‘pointed‐end actin filament capping’ (Figure 2C). GO analysis showed that the molecular function of highly expressed proteins was significantly enriched during ‘binding’ and ‘hydrolase activity’ (Figure 2C). Moreover, the pathway of synaptic vesicle cycle, endocytosis, Mammalian target of rapamycin (mTOR) signaling pathway phagosome, and cell cycle were concerned in plasma exosome‐treated microglia by the KEGG pathway analysis (Figure 2D).

Interestingly, V1G1 was downregulated in the PD‐exo‐treated microglia group. GO functional enrichment analysis found that lysosomal V1G1 molecules were mainly enriched under the biological function of vesicular proton transfer V‐type ATPase, which participated in the acidification of lysosome lumen and the regulation of pH value. KEGG pathway enrichment analysis found that lysosomal V1G1 molecules were enriched in the phagosome and mTOR signaling pathways, indicating that lysosomal V1G1 may be involved in the autophagy–lysosome pathway.

### 3.3 Lysosomal V1G1 expression was downregulated in MPTP‐induced PD models and PD‐exo‐treated BV2 cells

**FIGURE 3:**
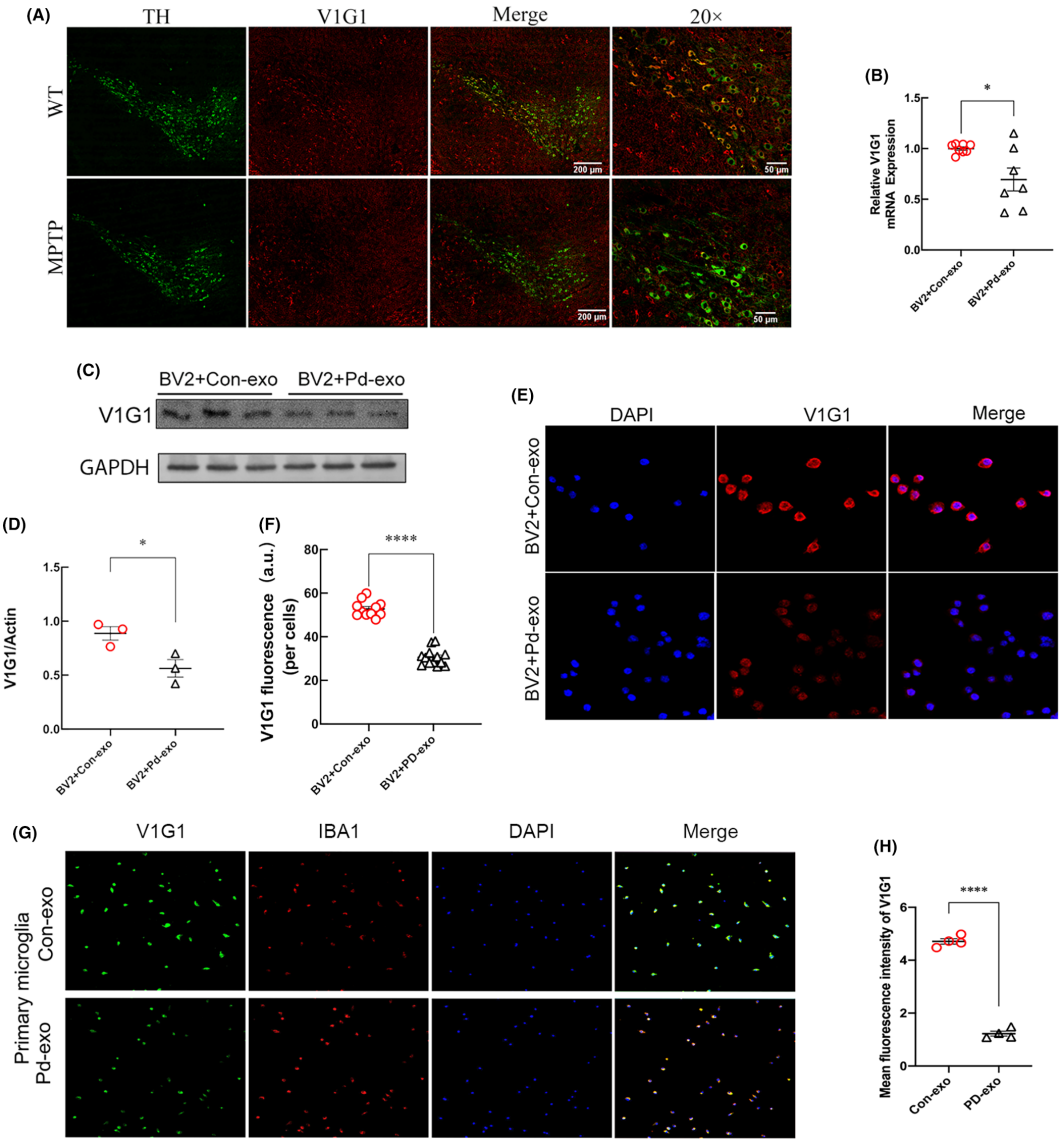
Lysosomal VIG1 was reduced in MPTP‐induced PD models and PD‐exo‐treated BV2 cells. (A) Representative images of double immunolabeling analysis for lysosomal V1G1 with either dopaminergic neuron marker TH in the SN of MPTP‐treated mice. (B) The mRNA levels of lysosomal V1G1 were detected by RT‐PCR in the PD‐exo‐treated microglia or Con‐exo‐treated microglia. (C, D) The lysosomal V1G1 was detected by western blot after the microglia were treated with PD‐exo or Con‐exo. The protein levels were normalized to β‐Actin. (E, F) Representative immunostaining and quantification of lysosomal V1G1 in the PD‐exo‐treated BV2 cells or Con‐exo‐treated BV2 cells. (G, H) Representative immunostaining and quantification of lysosomal V1G1 in the PD‐exo‐treated primary microglia or Con‐exo‐treated primary microglia. *p < 0.05, ****p < 0.0001.

To investigate whether lysosomal V1G1 was involved in the pathology of PD, the change of lysosomal V1G1 expression was evaluated in a PD mouse model induced by MPTP. Immunohistofluorescent staining showed that the number of TH+ dopaminergic neurons in the substantia nigra (SN) of the MPTP‐injected mice was reduced compared with wild‐type mice, indicating that the PD model had been established (Figure 3A). Moreover, the lysosomal V1G1 levels in the SN were noticeably lower in the MPTP than in the control group, suggesting an association between lysosomal V1G1 levels and incidence of PD.

To verify the results of proteomics and detect the expression of lysosomal V1G1 in cell models, the BV2 microglial cell line was treated with the same concentration of PD‐exo and Con‐exo for 24 h. Both real‐time quantitative polymerase chain reaction (RT‐qPCR) and western blotting analyses showed that the expressions of lysosomal V1G1 decreased in PD‐exo‐treated BV2 cells (Figure 3B–D). Similarly, immunofluorescence showed that the fluorescence intensity of lysosomal V1G1 in BV2 microglial cells treated with PD‐exo was lower than that of Con‐exo‐treated cells (Figure 3E,F). In primary microglia, the fluorescence intensity of lysosomal V1G1 decreased after PD‐exo treatment (Figure 3G,H). Thus, PD‐exo treatment may change the microglial lysosome function via downregulating lysosomal V1G1.

### 3.4 PD‐exo causes lysosomal impairment

**FIGURE 4:**
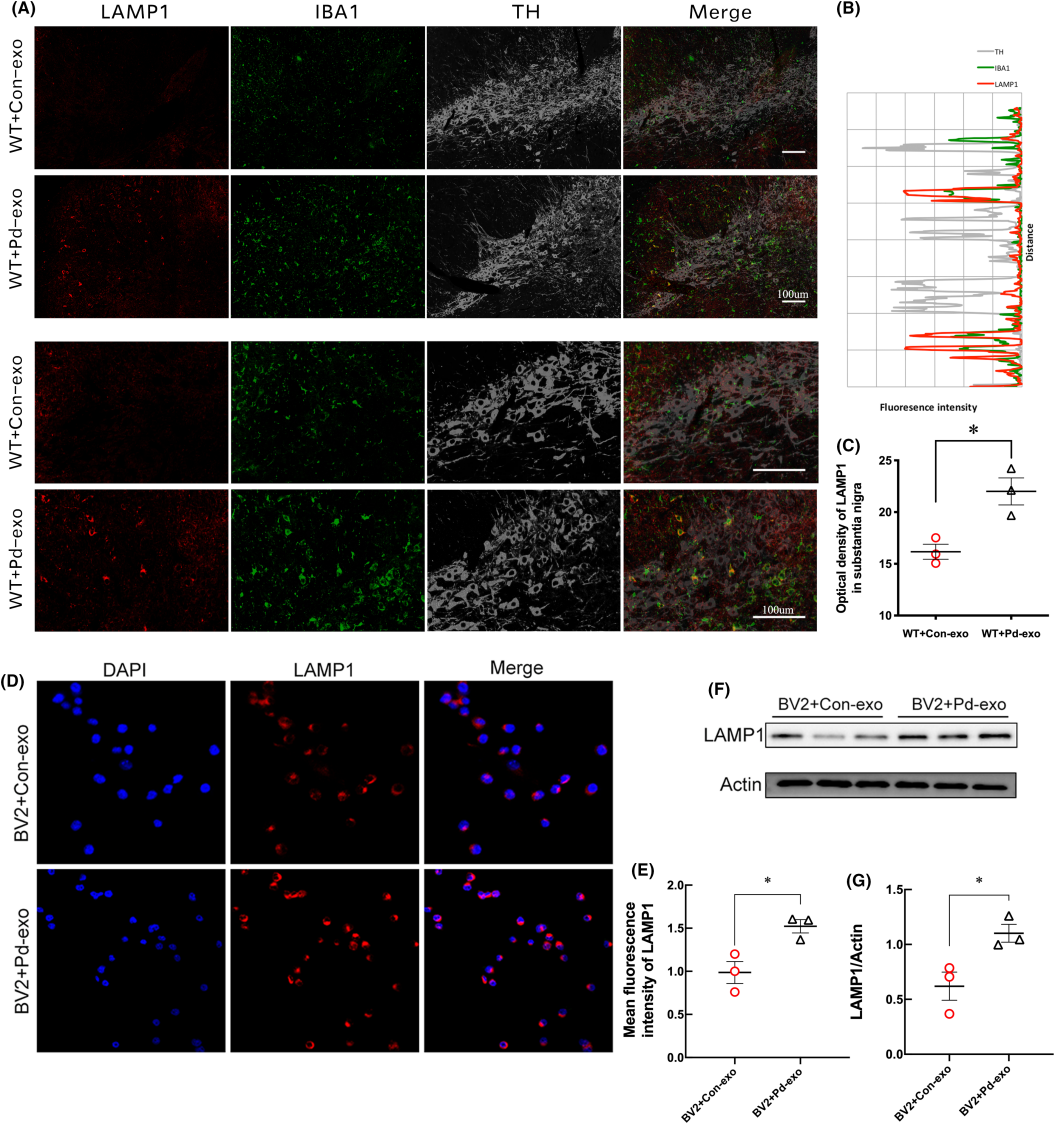
Treatment of PD‐exo increased the intensity of LAMP1 in microglia. (A) The triple‐stained image of LAMP1 (red) with IBA1 (green) and TH (gray) in the SN of PD‐exo‐treated mice or Con‐exo‐treated mice. (B) Representative fluorescence intensity profiles of LAMP1, IBA1, and TH in the partial regions of the high‐power magnification image. (C) The statistical graph of LAMP1 in the SN. (D) Representative double‐immunofluorescent staining of LAMP1 and DAPI and the statistical graph (E) of LAMP1 in the exosomes‐treated BV2 cells. (F) The representative western blot bands and the statistical graph (G) of LAMP1 protein expressions in the exosomes‐treated BV2 cells. *p < 0.05.

To determine the effect of PD‐exo on lysosomes, the change of lysosomal marker lysosomal‐associated membrane protein 1 (LAMP1) was evaluated in a PD mouse model treated by PD‐exo. Confocal immunofluorescence analysis revealed an increase in the intensity of the LAMP1 in microglia rather than in neurons (Figure 4A–C). A similar observation was made in BV2 microglial cells. Cell immunofluorescence experiments showed that the fluorescence intensity of LAMP1 in microglial cells was enhanced after PD‐exo treatment, indicating that the abundance of lysosomes increased (Figure 4D,E). Immunoblot analysis confirmed increased LAMP2 levels in PD‐exo‐treated BV2 cells (Figure 4F,G). The results suggest that PD‐exo downregulated the expression of lysosomal V1G1, thus increasing the number of lysosomes.

**FIGURE 5:**
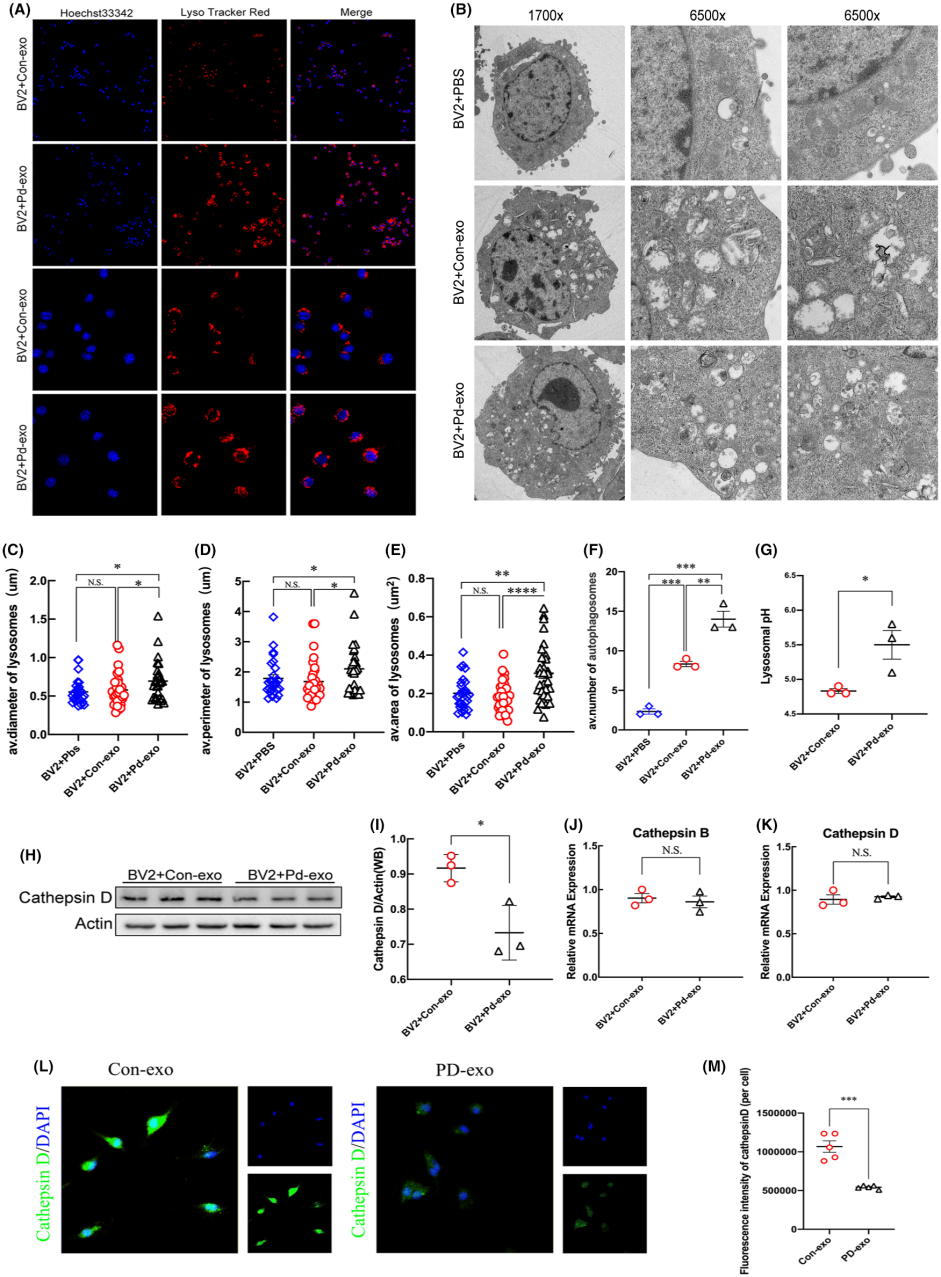
PD‐exo impaired lysosomal morphology and function. (A) Lyso‐Tracker probes tracked the dynamic changes of intracellular lysosomes after exosome intervention. (B) Morphological characteristics of intracellular lysosomes incubated with exosomes under transmission electron microscopy. (C) Quantification of lysosome diameter. (D) Quantification of lysosome perimeter. (E) Quantification of lysosome area. (F) Quantification of the number of autophagosomes. (G) Lysosomal pH measurements were determined for exosomes‐treated BV2 cells. (H) The representative western blot bands and the statistical graph (I) of cathepsin D protein expressions in the exosomes‐treated BV2 cells. The mRNA levels of cathepsin B (J) and cathepsin D (K) were detected by RT‐PCR in the PD‐exo‐treated microglia or Con‐exo‐treated microglia. (L, M) Representative immunostaining and quantification of cathepsin D in the PD‐exo‐treated primary microglia or Con‐exo‐treated primary microglia. *p < 0.05, **p < 0.01, ***p < 0.001, ****p < 0.0001.

Lyso‐Tracker Red is a lysosome‐specific probe that can pass through the cell membrane and be used for specific fluorescent staining lysosomes in living cells. The results showed that the number of lysosomes in microglia treated with Con‐exo was lower and mainly distributed on one side of the nucleus. After PD‐exo treatment, the number of lysosomes increased, and the distribution of lysosomes became distributed around the nucleus (Figure 5A). Next, the morphology of lysosome was observed by TEM. TEM analysis revealed that the lysosomes in microglia were swollen after the PD‐exo treatment, and the diameter, perimeter, and area of the lysosomes increased (Figure 5B–E). Moreover, the autophagosomes in microglia were aggregated, indicating lysosomal impairment in PD‐exo‐treated BV2 cells (Figure 5F).

To investigate the impact of PD‐exo on lysosomal function, the pH of lysosomes in BV2 cells was measured using LysoSensor Yellow/Blue dextran ratiometric dye. The results revealed that the pH of lysosomes in Con‐exo‐treated cells was 4.7, while the pH of lysosomes in PD‐exo‐treated cells was slightly above 5.5 (Figure 5G). The increase in lysosomal pH associated with PD‐exo is associated with disorders of lysosomal acidification owing to a defective proton pump.

Additional evidence that lysosomes in PD‐exo‐treated cells are not functioning properly came from an experiment where cathepsin D activity was examined. Despite lysosome accumulation, the activity of the lysosomal enzyme cathepsin D in PD‐exo‐treated cells significantly decreased relative to Con‐exo‐treated cells (Figure 5H,I). In primary microglia, the fluorescence intensity of cathepsin D also decreased after PD‐exo treatment (Figure 5L,M). However, qPCR analysis found that the messenger ribonucleic acid (mRNA) expression levels of cathepsin B and cathepsin D were not significantly changed (Figure 5J,K). The function of hydrolyzing protein should maintain a highly acidic environment in the lumen of lysosomes, where its hydrolase activity played an important role in degrading intracellular membranous organelles and large protein aggregates.

### 3.5 The degradation abilities of microglia are impaired under PD‐exo treatment

**FIGURE 6:**
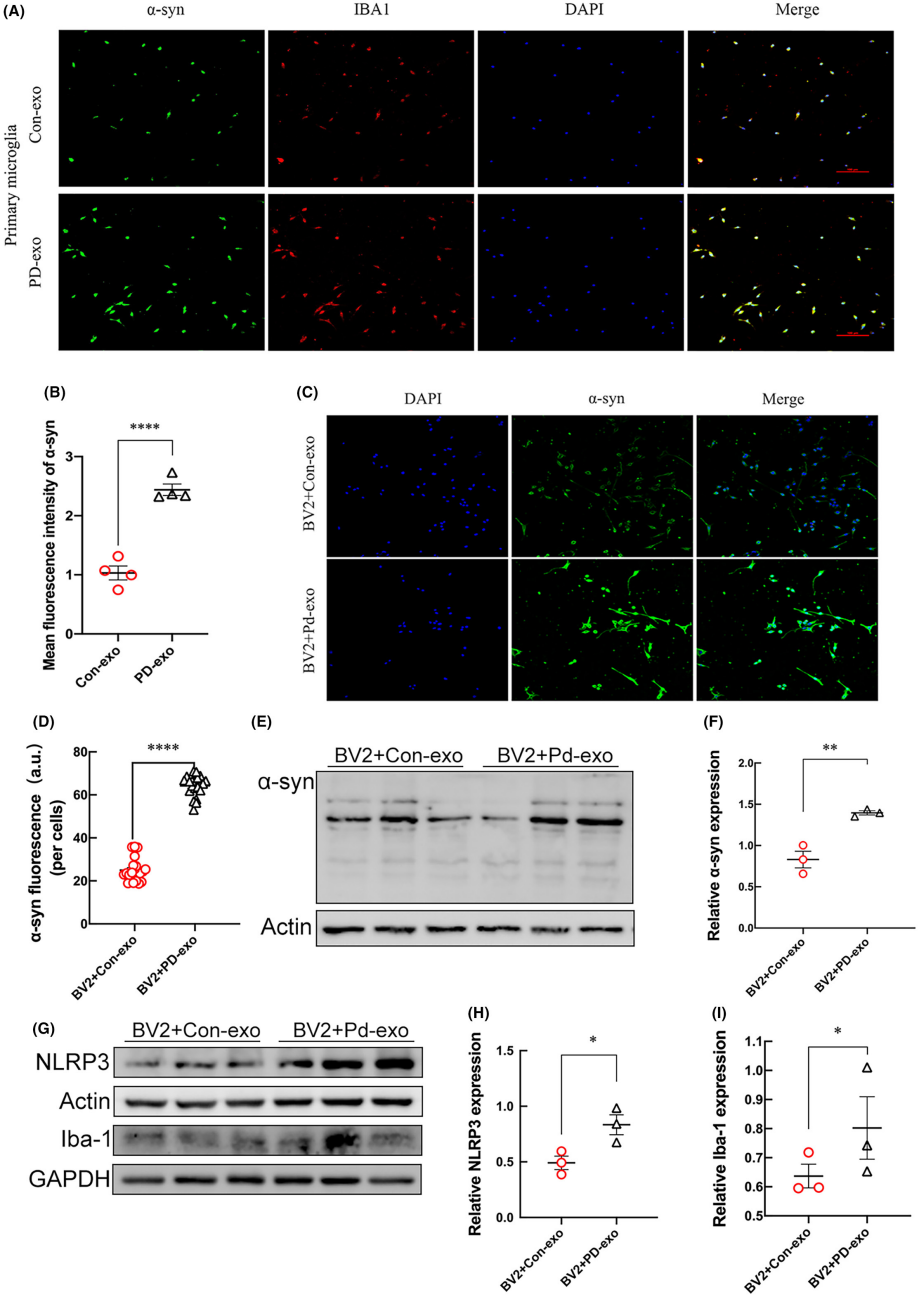
PD‐exo provokes α‐syn aggregation and inflammatory activation in microglia. (A) Representative triple‐immunofluorescent staining of α‐syn, IBA1, and DAPI and the statistical graph (B) of α‐syn in the exosomes‐treated primary microglia. (C) Representative double‐immunofluorescent staining of α‐syn and DAPI and the statistical graph (D) of α‐syn in the exosomes‐treated BV2 cells. (E) The representative western blot bands and the statistical graph (F) of α‐syn protein expressions in the exosomes‐treated BV2 cells. (G) The protein expressions of NLRP3 and Iba‐1 were detected by western blot after the microglia were treated with PD‐exo or Con‐exo. The quantitative data of NLRP3 (H) and Iba‐1 (I) protein expressions. *p < 0.05, **p < 0.01, ****p < 0.0001.

To investigate the potential function of the PD‐exo on the degradation of insoluble proteins and neuroinflammation, primary microglia were treated with Con‐exo and PD‐exo carried with aggregated α‐syn. After being treated for 24 h, the aggregation of α‐syn in microglia was detected by immunofluorescence. As shown, PD‐exo results in the aggregation of α‐syn in microglia (Figure 6A,B). In BV2 cells, the fluorescence intensity of α‐syn increased after PD‐exo treatment (Figure 6C,D). To further confirm which α‐syn form increased after PD‐exo treatment, western blotting was used to detect α‐syn changes in exosome‐treated BV2 cells. Compared with the Con‐exo treatment, intracellular α‐syn increased more after PD‐exo treatment, and the increased α‐syn was mostly in the form of oligomers (Figure 6E,F). PD‐exo impairs lysosomal protein degradation, which causes the accumulation of α‐syn.

Lysosome function is important to microglial biology given the critical role microglia play in clearing exogenous debris, such as pathologic α‐syn. Western blotting experiments showed that compared with Con‐exo treatment, PD‐exo‐treated microglia cells showed increased expressions of ionized calcium binding adaptor molecule 1 (Iba1) and NLR family pyrin domain containing 3 (NLRP3) inflammatory bodies (Figure 6G–I). The involvement of microglial neuroinflammation in the PD‐exo‐induced pathology is supported by the facts of NLRP3 inflammasome activation and overexpression of microglial marker Iba1.

### 3.6 Loss of lysosomal V1G1 alters the degradation of α‐syn by impairing lysosomal function

**FIGURE 7:**
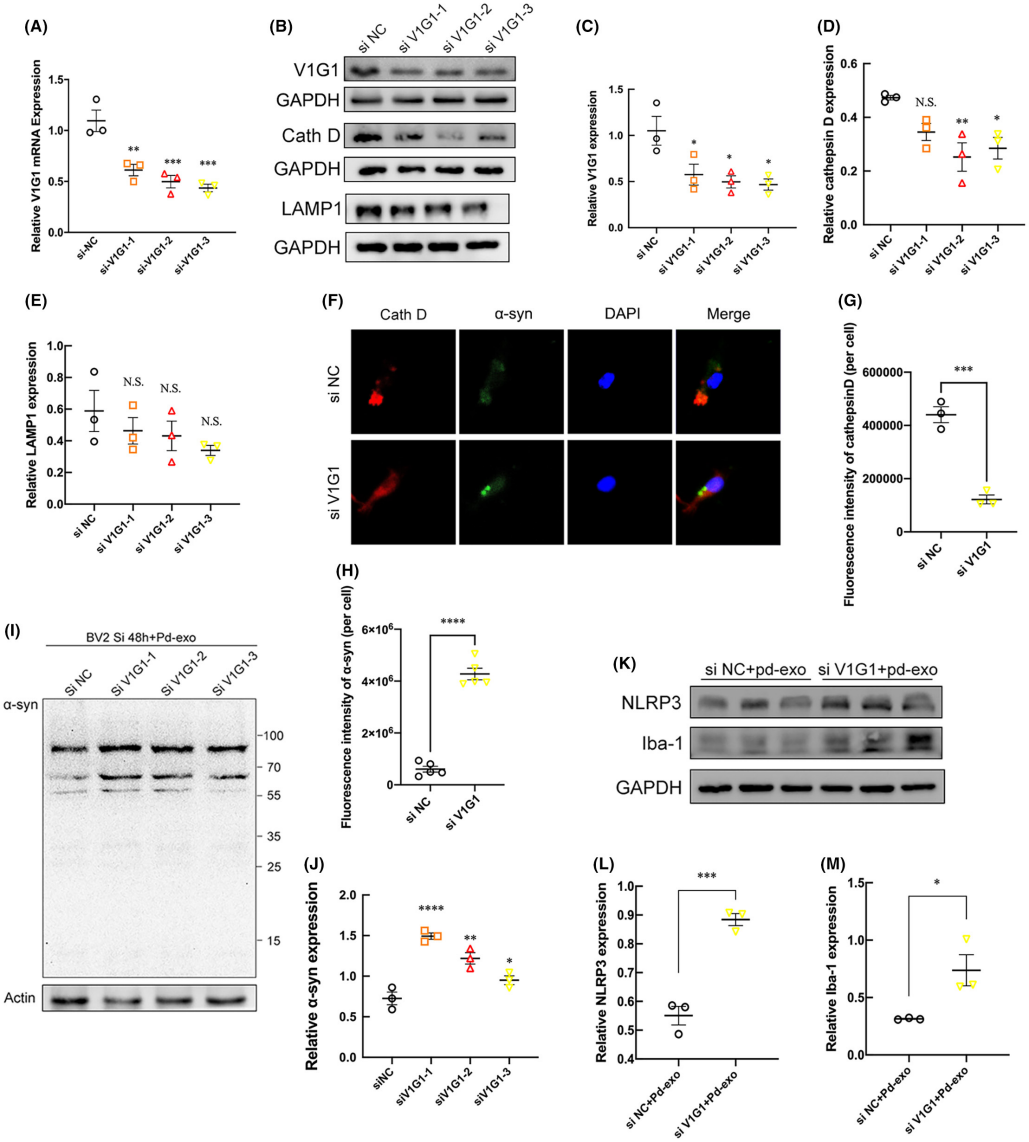
Knockdown of lysosomal V1G1 impairs lysosomal function, leading to α‐syn accumulation and enhanced inflammation. Detection of lysosomal V1G1 knockdown by RT‐qPCR (A) and western blotting (B, C). (B) The protein expressions of cathepsin D and LAMP1 were detected by western blot in the lysosomal V1G1‐knockdown microglia. The quantitative data of cathepsin D (D) and LAMP1 (E) protein expressions. (F) Representative triple‐immunofluorescent staining of α‐syn, cathepsin D, and DAPI and the statistical graph of cathepsin D (G) and α‐syn (H) in the siRNA‐treated primary microglia. (I) The representative western blot bands and the statistical graph (J) of α‐syn protein expressions in the lysosomal V1G1‐knockdown microglia. (K) BV2 cells were pretreated with control or V1G1 siRNA, then incubated with PD‐exo for 24 h. The representative western blot bands and the statistical graph of NLRP3 (L) and Iba‐1 (M) protein expressions. *p < 0.05, **p < 0.01, ***p < 0.001, ****p < 0.0001.

To confirm whether changes in lysosomal V1G1 expression would affect the degradation of exosomal α‐syn, three small interfering RNAs were used to knockdown the expression of V1G1 in BV2 cells, and the effect of V1G1 knockdown was verified. The qPCR results showed that the three small interfering RNAs (siRNAs) could successfully knockdown V1G1 at the mRNA level (Figure 7A). Western blotting further verified the knockdown of V1G1 at the protein level (Figure 7B,C).

To determine the lysosomal function under the knockdown of V1G1, the expression of cathepsin D and LAMP1 was examined by western blotting. Immunoblotting analysis revealed a decrease in the lysosomal enzyme cathepsin D in V1G1 knockdown BV2 cells over control cells (Figure 7B,C). However, the intensity of lysosomal marker LAMP1 did not change with the knockdown of V1G1 (Figure 7E). The results demonstrate that a significant portion of LAMP1‐labeled organelles lacks major lysosomal hydrolases. In primary microglia, the fluorescence intensity of cathepsin D also decreased after the knockdown of V1G1 (Figure 7F,G). As shown, the knockdown of V1G1 results in the aggregation of α‐syn in microglia (Figure 7F,H).

After knocking down lysosomal V1G1 with small interfering RNA for 48 h, the same concentration of PD‐exo was added to BV2 cells for 12 h. Western blotting results showed that the content of α‐syn in cells increased, indicating that the ability of lysosomes to degrade α‐syn reduced after V1G1 knockdown, resulting in an increased accumulation of α‐syn in cells (Figure 7I,J). Consistently, increased accumulation of α‐syn induces activation of Iba1 and NLRP3 inflammatory bodies (Figure 7K–M).

### 3.7 Lysosomal V1G1 overexpression rescues MPTP neurotoxicity in mice

**FIGURE 8:**
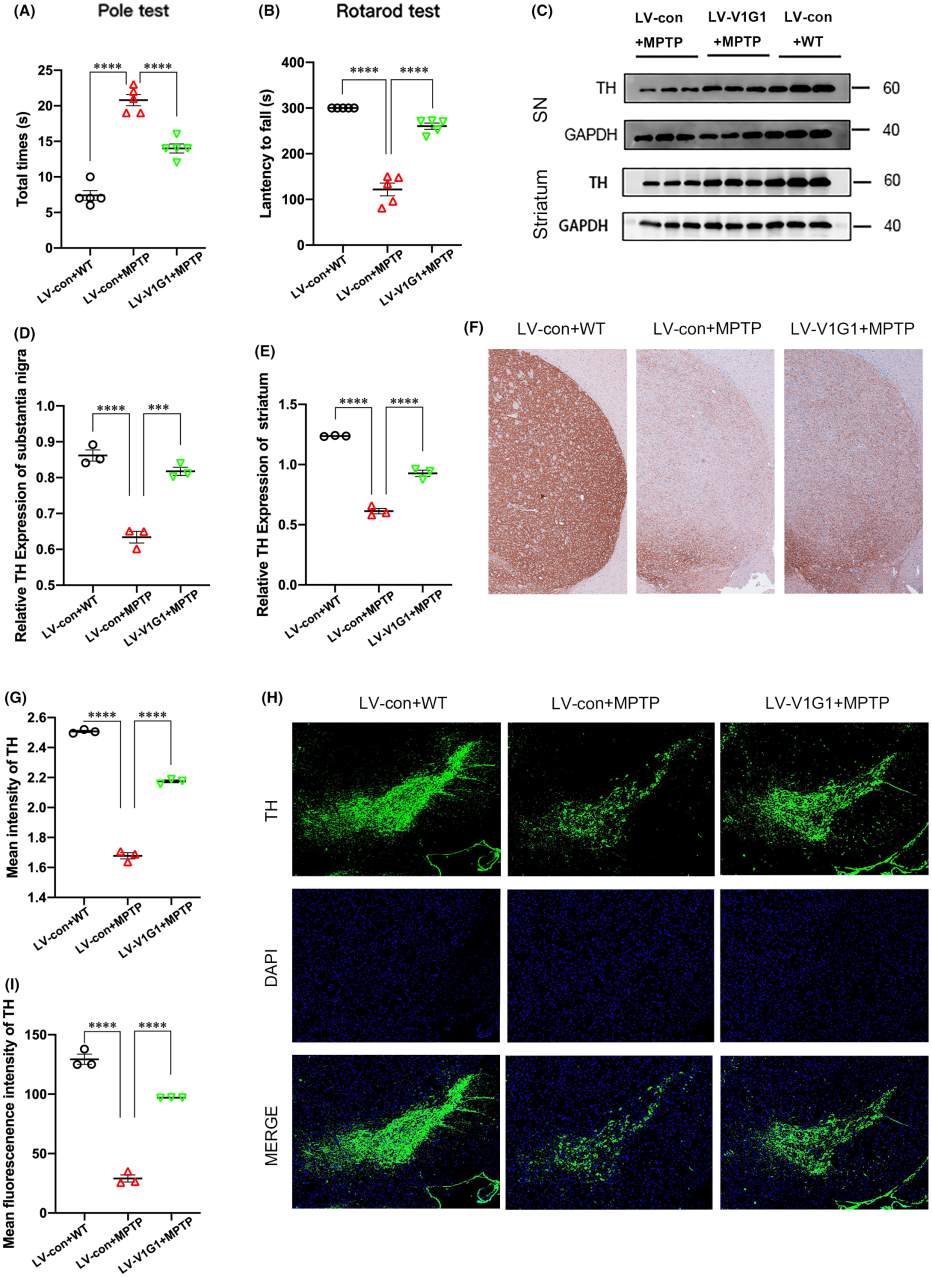
Lysosomal V1G1 overexpression attenuates MPTP neurotoxicity in vivo. Lentiviral particles containing the control or V1G1 complementary deoxyribonucleic acid were injected unilaterally into the striatum. After 1 week, the mice in the MPTP group were continuously intraperitoneally administered with MPTP solution for 7 days, while the mice in the control group were administered with an equal volume of saline. (A, B) Behavioral tests include the pole test and rotarod test. (C–E) Western blot analysis and quantification of TH in the ipsilateral substantia nigra and striatum. (F, G) Representative images and quantification of TH‐positive terminals in the ipsilateral striatum. (H, I) Representative images and quantification of TH‐positive neurons in the ipsilateral substantia nigra pars compacta. ***p < 0.001, ****p < 0.0001.

Lentiviral particles containing the control or V1G1 complementary deoxyribonucleic acid were injected unilaterally into the striatum. After 1 week, the mice in the MPTP group were continuously intraperitoneally administered with MPTP solution for 7 days, while the mice in the control group were administered with an equal volume of saline. The motor function of MPTP‐ or saline‐treated mice was measured on days 6–7 after the last injection. In comparison with that of the control group, the pole test indicated a significantly prolonged time in the MPTP group (Figure 8A). Interestingly, V1G1 overexpression partially reversed these effects (Figure 8A). In the rotarod test, the average latency on the rotated rod decreased after MPTP treatment in wild‐type (WT) mice but not in mice with V1G1 overexpression (Figure 8B). These data suggested that V1G1 overexpression protects mice from MPTP‐induced motor function deficits.

The function of the nigrostriatal dopaminergic pathway was examined by immunoblotting and immunostaining for tyrosine hydroxylase (TH). Immunoblotting showed that MPTP administration significantly reduced the TH protein levels in the SN and striatum, which was partly prevented in mice with V1G1 overexpression (Figure 8C–E). As expected, immunohistochemical analysis showed that dopaminergic neurons in the striatum in the MPTP group were severely damaged in comparison with those in the control group, but this effect was reversed by V1G1 overexpression (Figure 8F,G). Similarly, MPTP administration led to the obvious loss of dopaminergic neurons in the SN in WT mice (Figure 8H,I). However, limited neuronal loss was noted in mice with V1G1 overexpression after MPTP treatment (Figure 8H,I). These data demonstrated the neuroprotective effects of V1G1 overexpression against MPTP‐induced nigral neuronal death in vivo.

## 4 DISCUSSION

**FIGURE 9:**
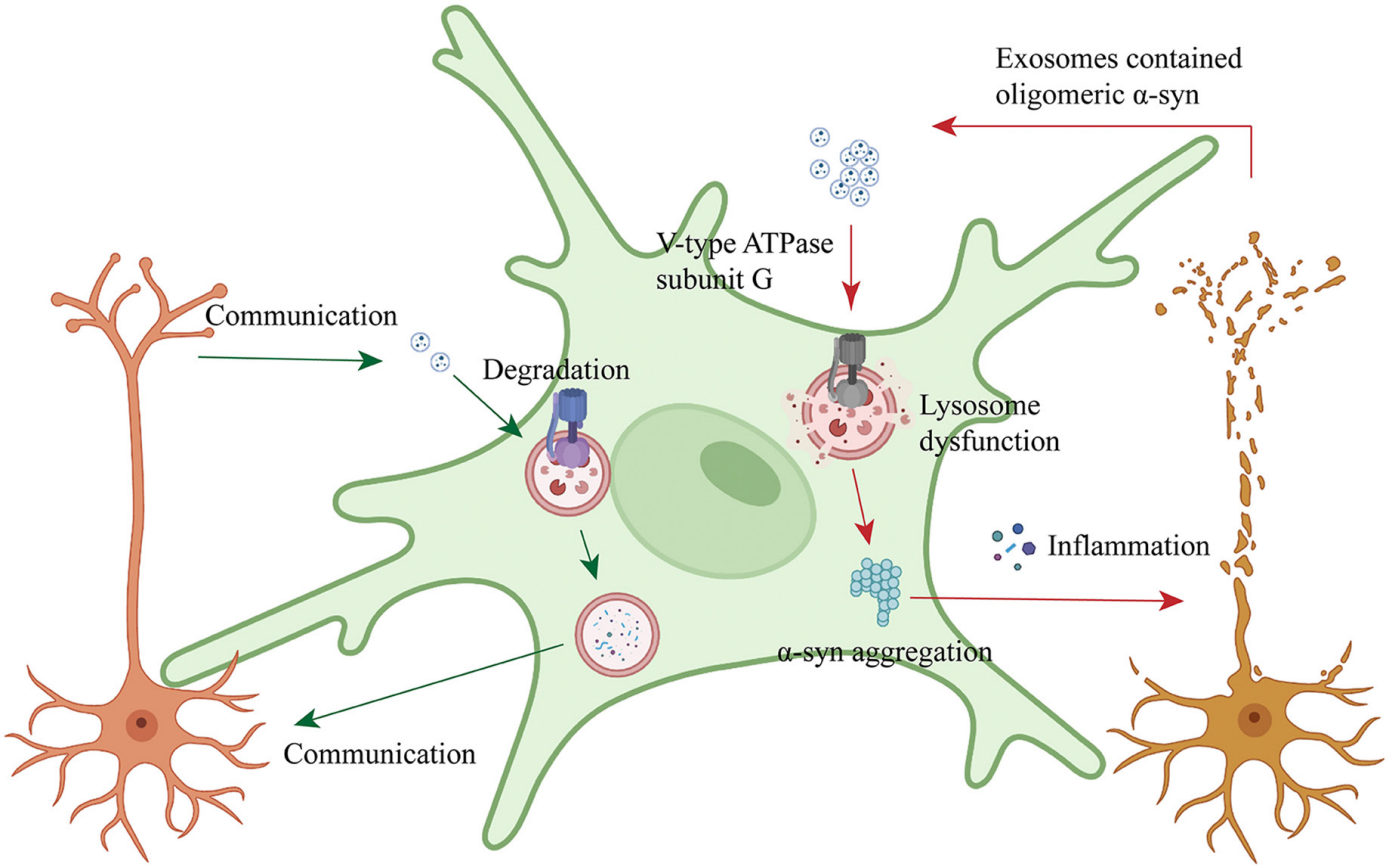
Plasma exosomes impair microglial degradation of α‐synuclein through V‐ATPase subunit V1G1. Plasma‐derived exosomes from patients with PD contain toxic α‐syn and PD‐exo downregulates lysosomal V1G1 expression; PD‐exo alternated the amount, morphology, and distribution of lysosomes in microglia and impaired the activity of hydrolytic enzymes in the lumen; PD‐exo augmented the accumulation of pathological α‐syn within microglia and induced NLRP3‐mediated neuroinflammation; and V1G1 deficiency impaired lysosomal function, leading to further aggregation of α‐syn in microglia and inflammation.

In this study, we showed that plasma‐derived exosomes from patients with PD contain more α‐syn, and PD‐exo downregulates lysosomal V1G1 expression; PD‐exo alternated the amount, morphology, and distribution of lysosomes in microglia, and impaired the activity of hydrolytic enzymes in the lumen; compared to Con‐exo treatment, PD‐exo augments the accumulation of pathological α‐syn within microglia and induces NLRP3‐mediated neuroinflammation; deficiency of V1G1 impairs lysosomal function, leading to further aggregation of α‐syn in microglia and activation of inflammation; and MPTP‐induced motor disturbance and neuronal toxicity in the SN and striatum were significantly alleviated in mice overexpressing V1G1. These findings suggest that V1G1 is involved in the effect of PD‐exo on microglial lysosomes and the degradation of α‐syn in PD‐exo by lysosomes (Figure 9). A better understanding of V‐ATPase, its subunits, and lysosomal function facilitates mechanistic research and therapeutic perspectives of PD.

The major finding of the study is the identification of lysosomal V1G1 as an essential molecule for digesting extracellular exosomal α‐syn via regulating lysosomal function. Lysosomal V1G1 is a subunit of lysosome V‐type ATPase, which maintains the intraluminal pH of the lysosome by a proton pump.^16^ We found that exosomal α‐syn derived from patients with PD decreases the protein levels of the lysosomal V1G1 in microglia. Remarkably, the low expression of lysosomal V1G1 contributes to α‐syn accumulation by disrupting lysosomal enzyme activity, which is dependent on the acidic environment of the lysosomal lumen. As pathogenic protein accumulation is a key feature in PD, compromised V‐type ATPase activity might participate in PD pathogenesis.

The accumulation of α‐syn and activation of microglia are the main pathological characteristics of α‐synucleinopathies. Although α‐syn initially accumulates in neurons, pathological transmission of α‐syn and nerve cell death exposes these proteins to microglia, which are responsible for removing misfolded and aggregated proteins from the brain.^17^, ^18^ Recent studies have shown that α‐syn can spread among neurons through the release of exosomes.^19^, ^20^ Moreover, exosomes derived from the CSF of patients with PD with dementia with Lewy bodies can promote the oligomerization of soluble α‐syn in neurons in the injected brain area of mice.^21^ This experiment found that compared with healthy controls, PD‐exo contained more pathological α‐syn, which is consistent with the conclusions of existing studies. Notably, microglia clear pathological α‐syn and regulate the dissemination of exosomal α‐syn.^22^ Moreover, exosomes from microglia contain α‐syn, which promotes the accumulation of α‐syn in neurons.^4^, ^5^ Recent studies have found that α‐syn preformed fibril induces an increase in the level of pellino E3 ubiquitin protein ligase 1 (PELI1) in microglia, and mediates the degradation of LAMP2 through the ubiquitin–proteasome system, resulting in increased autophagosome–multivesicular body fusion, which eventually leads to the secretion of exosomes and exosomal α‐syn.^5^ These results identify a connection between exosome metabolism, lysosomal activity, and the aggregation and degradation of α‐syn with unique characteristics.

Given that lysosomal function is necessary for the degradation of pathogenic proteins, including α‐syn, and abnormal lysosomal function occurs in the brains of patients with PD, the importance of lysosomal pH homeostasis becomes increasingly recognized.^23^, ^24^, ^25^ The function of hydrolytic enzymes in the lysosomal lumen is highly dependent on the high acidic pH value, and the V‐type ATPase proton pump on the lysosomal membrane is the key molecule to maintain lysosomal acidification.^26^ Previous reports have shown that impairment of V‐type ATPase function is associated with inherited PD, such as mutations of ATP6AP2 and ATP13A2.^27^, ^28^, ^29^, ^30^ Recently, *LRRK2*, the virulence gene of PD, was associated directly with the a1 subunit of V‐ATPase, and mutations of *LRRK2* resulted in lysosomal expansion and diminished lysosomal degradation of substrates.^31^ Moreover, Rab‐interacting lysosomal protein regulates the activity of V‐type ATPase by directly interacting with lysosome V1G1, indicating that lysosome V1G1 plays an indispensable role in the function of V‐type ATPase.^11^ Furthermore, lysosome V1G1 also plays a role in lysosome production.^12^ To our knowledge, this is the first study to report that incubation with PD‐exo downregulates the expression of lysosomal V1G1. Importantly, PD‐exo impairs lysosomal function and affects lysosomal morphological distribution. This may represent that the downregulation of V1G1 can affect the function of lysosomes and impair the clearance of pathological α‐syn by lysosomes. Indeed, after we knocked down the expression of V1G1 with siRNA, the decreased α‐syn clearance resulted in increased intracellular α‐syn aggregation. This provides an explanation for how PD‐exo contributes to the progress of PD.

Lysosomal processing capacity gradually decreases with age, and the correlation of lysosomal dysfunction with PD pathogenicity has been supported by evidence from genetic studies. For example, pathogenic variants in *GBA* confer a higher risk of developing PD, and mutations in the *ATP13A2* gene encoding the lysosomal ATPase cause juvenile‐onset familial PD.^32^ Recently, genome‐wide association studies have also identified the lysosomal genes *CTSD*, *CTSB*, *ATP6V0A1*, *NEU1*, and *SLC17A5* as PD risk genes.^33^, ^34^, ^35^ Common and rare variants in lysosomal genes may contribute to lysosomal dysfunction and, thus, affect PD susceptibility. Mutations in *GBA* gene may lead to loss of GCase activity and lysosomal dysfunction, thereby impairing α‐syn degradation. Studies in mouse models suggest that AAV9 vector‐based gene therapies designed to deliver functional *GBA1* genes to the brain may slow or halt disease progression.^36^ Increased LRRK2 kinase activity may impair the function of lysosomes and contribute to PD pathogenesis. Preclinical and clinical data revealed that small molecule inhibitors of LRRK2 kinase have the potential to correct lysosomal dysfunction in patients with PD.^37^ Regulating the function of V‐ATPase may affect lysosomal activity, thereby affecting acidification and multiple signaling pathways in particular cells. This study shows that regulating the function of V‐ATPase affects lysosomal activity and α‐syn metabolism, and is the first to demonstrate the protective effect of regulating V‐ATPase on PD pathology. The application of nanomaterials is also an interesting way to stimulate lysosomal function, and multiple studies have shown that these drugs can reacidify lysosomal pH in cultured cells.^38^, ^39^ Exploring the therapeutic implications of modulating lysosomal pH for neurodegenerative diseases would be interesting.

Targeting lysosomal acidification and V‐ATPase provides therapeutic benefits in neurodegenerative diseases, such as AD.^40^, ^41^, ^42^ However, the regulated role of V‐ATPase in PD needs further investigation. In this study, V1G1 and lysosomes were involved in the removal of exosomal α‐syn by microglia, and in turn, α‐syn could also affect the function of lysosomes. Therefore, targeting V1G1 molecules, enhancing lysosomal function, or promoting the autophagy‐lysosomal pathway is expected to increase the clearance of pathological α‐syn by microglia, thereby reducing the dissemination of α‐syn and the progression of PD. Although it is generally accepted that the multi‐subunit V‐ATPase proton pump is imperative for maintaining lysosomal acidification and function, further understanding of the subunit diversity that regulates V‐ATPase in the CNS may help identify the therapeutic potential for neurodegenerative diseases.

## AUTHOR CONTRIBUTIONS

Experiments were designed by Y.N.L., Y.X., and T.W. Experiments were performed by Y.N.L., Y.X., S.‐J.Y., X.‐S.C., L.K., Y.‐D.S., Y.‐M.W., W.‐K.Z., and T.W. Data were interpreted by Y.‐M.W., Y.N.L., J.‐W.W., Z.‐J.J., Q.‐L.Z., Y.X., and T.W. The manuscript was written by Y.‐M.W., Y.N.L. and Y.X.

## CONFLICT OF INTEREST STATEMENT

The authors declare no competing interests.

## Supporting information


Figure S1.–S8.

## Data Availability

The authors declare that when there is a reasonable request, the authors shall request all the data contained in this study.
