# Supplementary material for: Plasma exosomes impair microglial degradation of α‐synuclein through V‐ATPase subunit V1G1
Source: CNS Neurosci Ther. 2024 May 3;30(5):e14738. doi: 10.1111/cns.14738 (PMC11069054; doi:10.1111/cns.14738)

Figure 1C

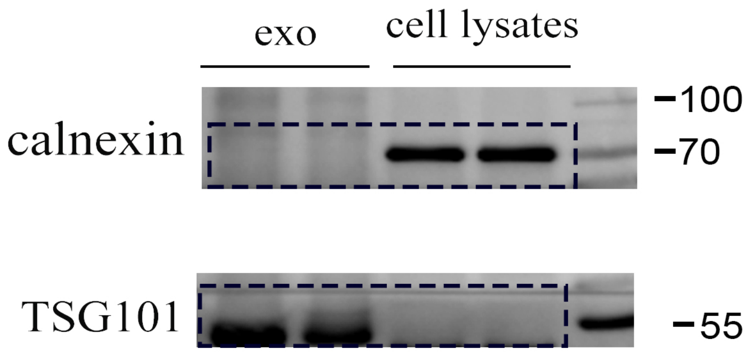

Figure 1F

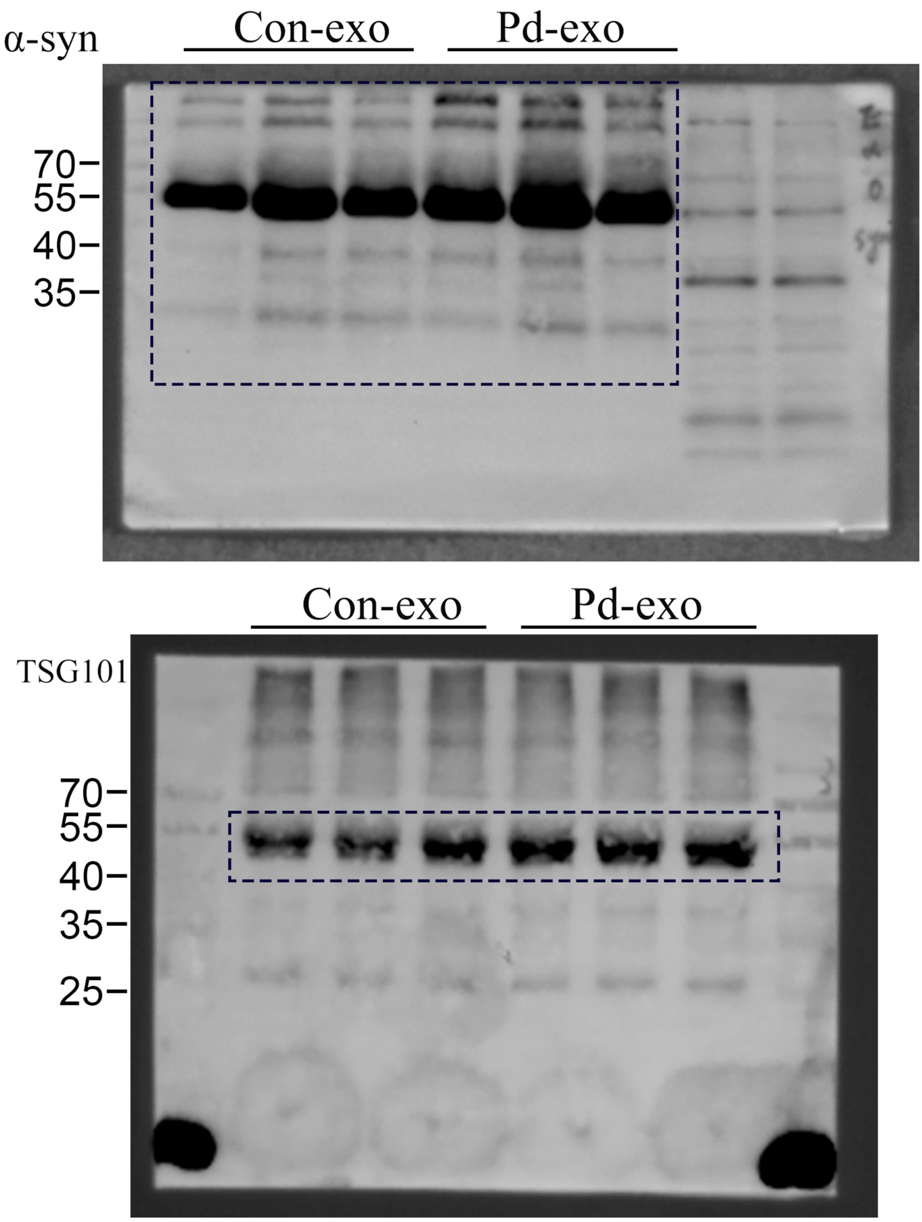

Figure 3C

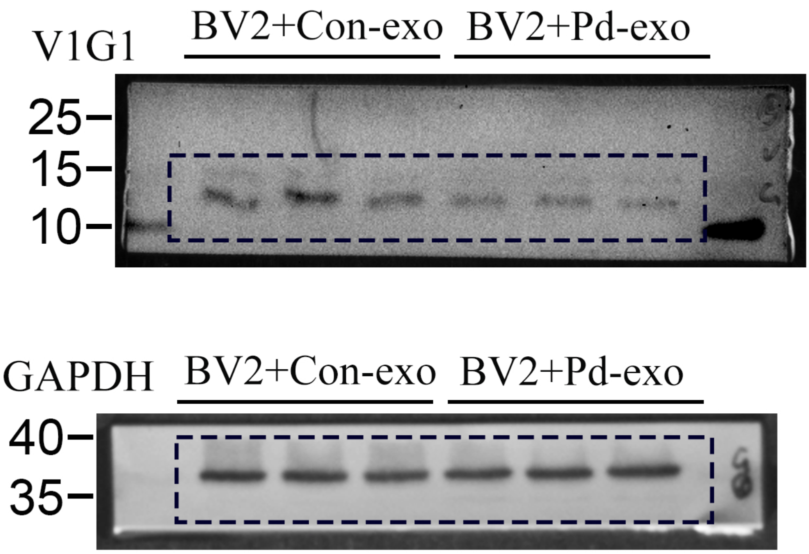

Figure 4F

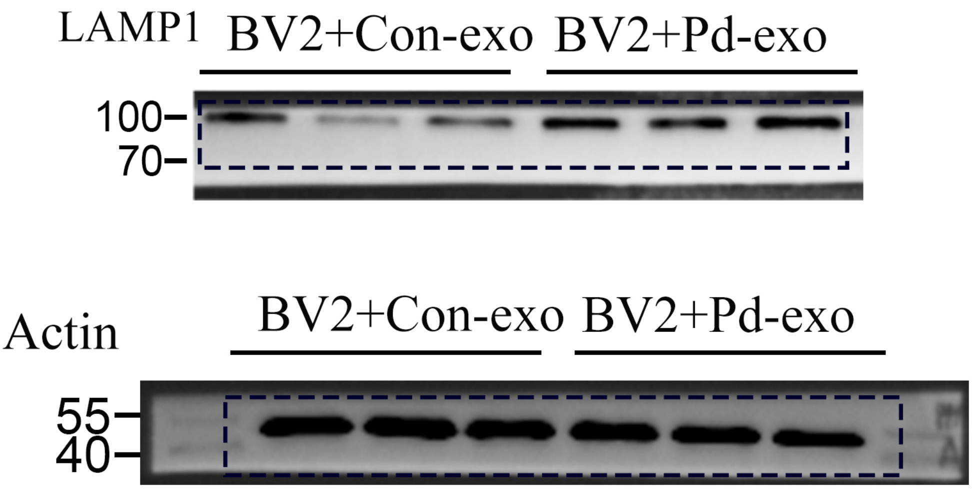

Figure 5H

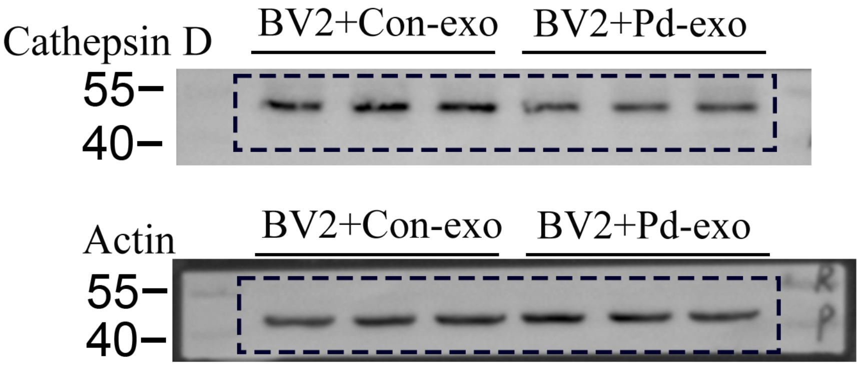

Figure 6E

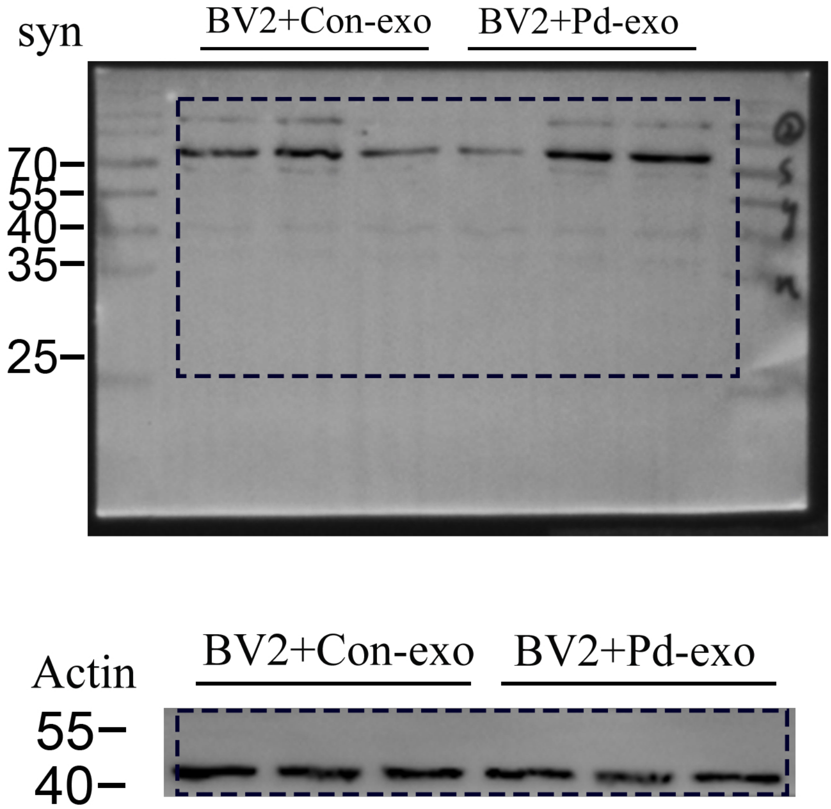

Figure 6G

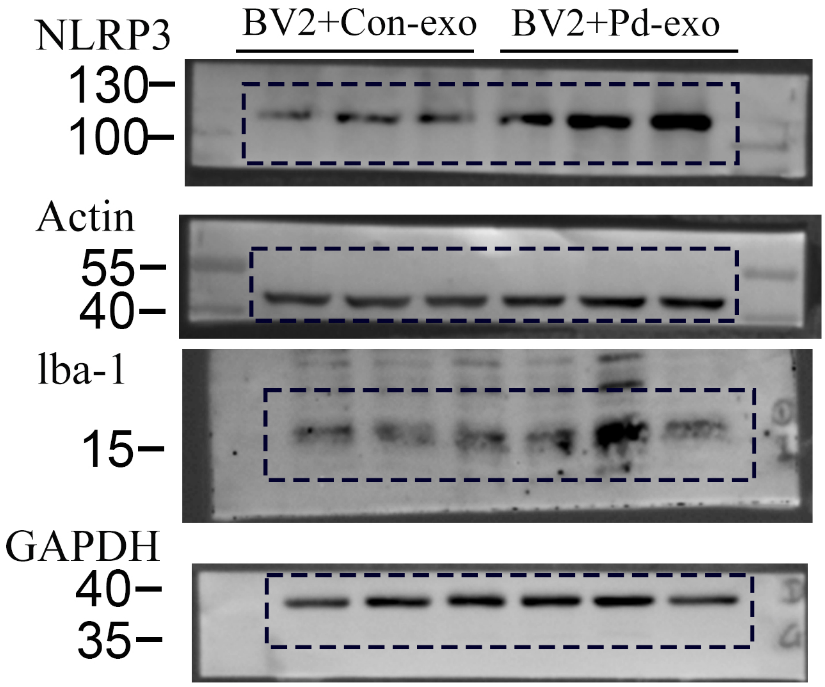

Figure 7B

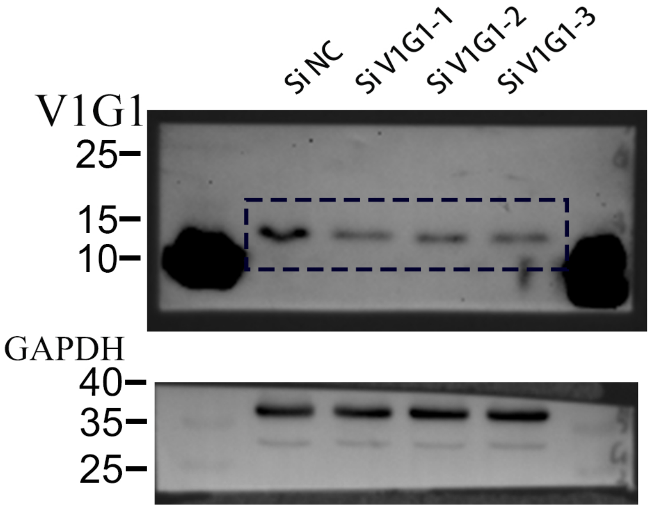

Figure 7B

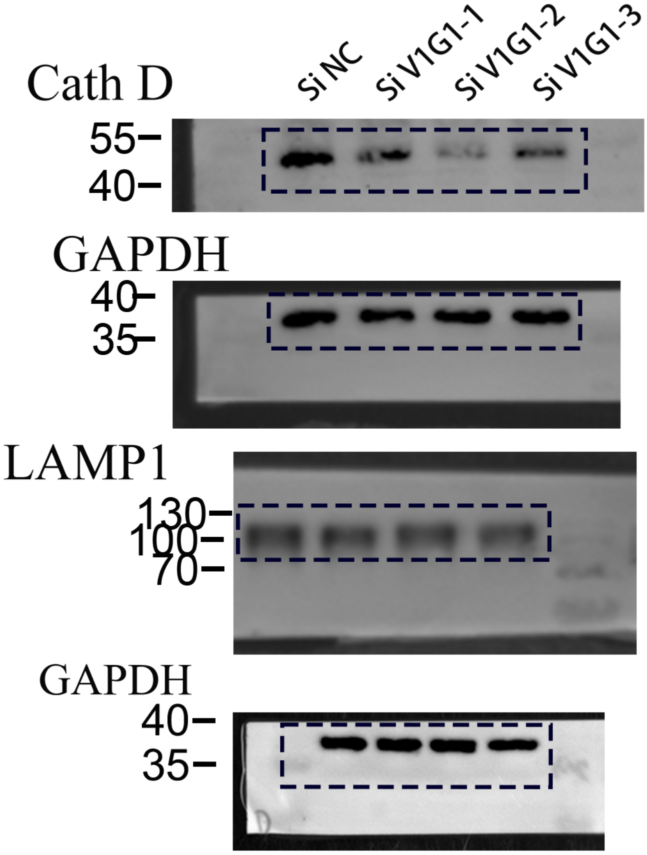

Figure 7I

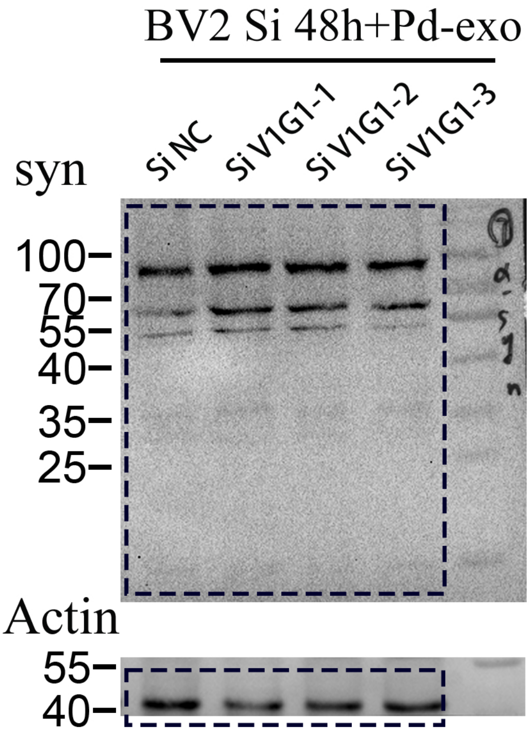

Figure 7K

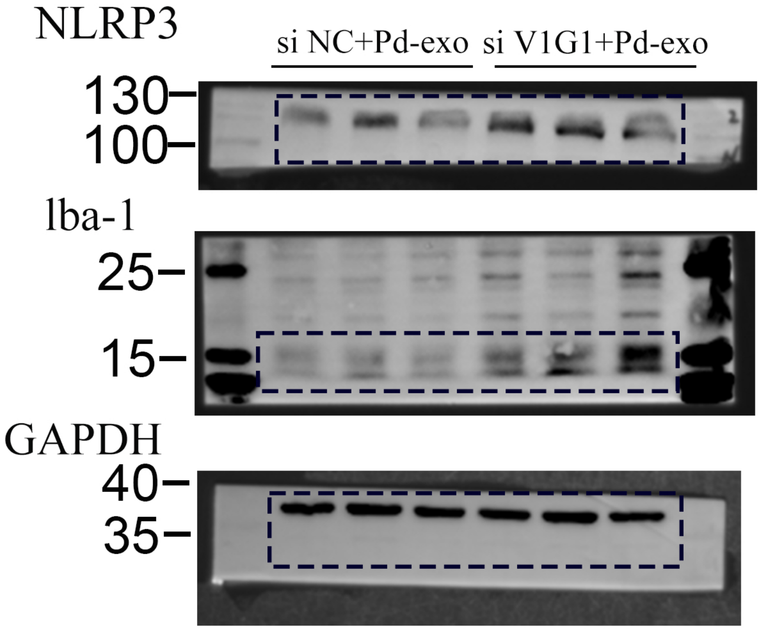

Figure 8C

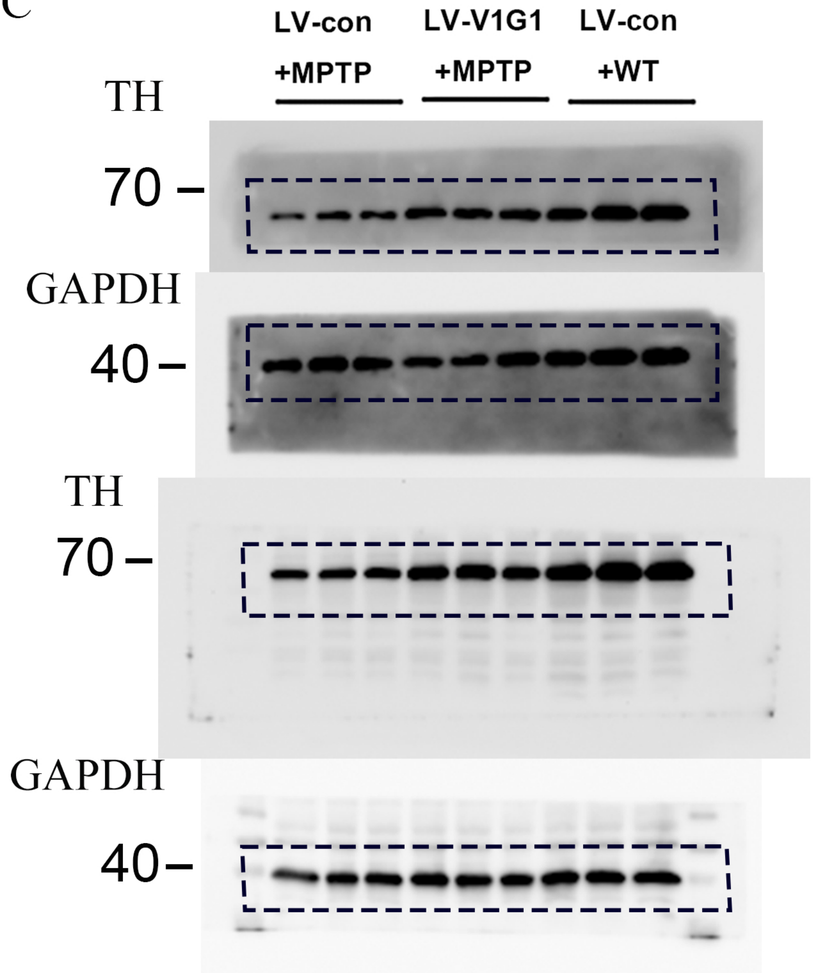

Supplement: Supplementary file 1 — Figure S1.–S8. [file CNS-30-e14738-s001.pdf]
